# Molecular profile PCR array of regenerative therapy (PRP, PRF& CpG-ODN) in wound healing of diabetic dogs

**DOI:** 10.1186/s12917-025-04892-9

**Published:** 2025-07-07

**Authors:** Olla A. Khalifa, A. M. Alakraa, A. H. Elkasapy

**Affiliations:** 1 Genetics and Genetic Engineering, Department of Animal Wealth Development, Faculty of Veterinary Medicine, Benha University, Benha, 13736 Egypt; 2 Department of Surgery, Anesthesiology and Radiology, Faculty of Veterinary Medicine, Benha University, Benha, 13736 Egypt

**Keywords:** PCR array, PRP, PRF, CpG-ODN, Diabetes, Dog

## Abstract

This study’s aim is to validate differential wound healing genes expression in canine with diabetes using regenerative medicine such as PRP, PRF, and CpG oligodeoxynucleotides (ODN), for these purpose thirty six diabetic dogs were used and subdivided into 6 groups (6 dogs each) with full-thickness incisional wounds, wound healing was evaluated with a focus on histological changes in wound tissue and gene expression after 3 weeks. The molecular mechanism of wound healing responding to different treatments in dogs were done using the new molecular technique RT2 Profiler PCR array (Qiagen), which enables us to view a focused panel of genes (84 genes) responsible for wound healing. CpG-ODN contributes to low cellular infiltration; PRP improves diabetic wound closure rapidly. The study illustrated that the healing rate was higher in the PRP & CpG-ODN. The animal in this group showed a clinically higher contraction rate of the wound area, upregulates 37 genes responsible for wound healing and downregulates ten genes for the same purpose, also marked collagen production were observed with complete epithelialization. Our study concluded that PRP & CpG-ODN was superior to PRF & CpG-ODN, PRP, PRF, CpG-ODN, and the control group, respectively, for enhancing wound healing in diabetic dogs.

**Supplementary Information:**

The online version contains supplementary material available at 10.1186/s12917-025-04892-9.

## Introduction

Diabetes mellitus is considered a serious factor that delays wound healing. This is accomplished due to vascular diminish, metabolic reasons, and weight loss. The healing of wounds denotes a dynamic, highly organized set of intricate actions [1]. It is characterized by three interrelating stages: inflammation, tissue formation, and tissue remodeling. This series of events leads to the formation of scar tissue, which is characterized by a smooth, glabrous epidermis that covers non-functional dermal tissue [2]. Regenerative medicine primarily aims to introduce treatments that help treat scarring, fibrosis, inflammation, as well as other detrimental effects in diseased or damaged animal tissues. Such treatments provide one component or more important for tissue regeneration (scaffold, growth factors, and cells) [3].

Platelet-rich plasma (PRP), or autologous conditioned plasma, is plasma with more platelets than the percentage that is usually found in the blood [4]. It was found to cause considerable changes in severe wound healing [5] and enhance recovery [6] and have an antimicrobial activity [7]. PRP is a growth factor with chemotactic and mitogenic characteristics. It possesses a complete complement of coagulation and growth factors, as well as a high level of platelets. It serves as a drug delivery system and tissue sealant, as the platelets initiate wound healing by releasing locally acting growth factors through α-granule degranulation [8]. The secretory proteins that are involved in the α-granules of platelets include platelet-derived growth factor (PDGF-AA, BB, and AB isomers), thrombospondin-1 (TSP-1), transforming growth factor-β (TGF-β), interleukin-1 (IL-1), fibronectin (Fn), platelet factor 4 (PF4), vascular endothelial growth factor (VEGF), fibrinogen (Ff), osteocalcin (Oc), platelet-derived angiogenesis factor (PDAF), osteonectin (On), epidermal growth factor (EGF), platelet-derived endothelial growth factor (PDEGF), epithelial cell growth factor (ECGF), and insulin-like growth factor (IGF). They facilitate the healing process by attracting undifferentiated cells to the newly formed matrix and thereby initiating cell division [9].

Platelet-rich fibrin (PRF) denotes a second‐generation platelet concentration that includes growth factors and platelets without using anticoagulants [10], nor blood biochemical manipulation and with a centrifugation cycle, so considered low in cost compared to recombinant growth factors/ hormones or other regenerative modalities [11]. It supports hemostasis. It releases cytokines and growth factors while remodeling tissues to enhance the recovery of wounds [12], such as VEGF, TGF-β1, EGF, interleukin-1β (IL-1β), IL-4, IL-6, and also tumor necrosis factor- α (TNF-α) [13].

Toll-like receptor-9 (TLR-9) signaling is activated by synthetic oligodeoxynucleotides (CpG-ODN), which express unmethylated motifs similar to bacterial DNA [14]. TLRs represent a group of molecules that identify invading microbes or viruses and then activate immune cell responses [15]. Activating the innate immune system helps heal wounds [16], as stimulation of TLRs accelerates this process [14]. Recent studies prove that CpG-ODN enhance the healing of skin wounds [14, 17].

CpG-ODN promote fibroblast proliferation and induce collagen production, an abundant ECM protein. Thus, they can enhance the healing of wounds [18]. Moreover, they stimulate activating macrophages and producing cytokines [19] and improve the ingestion of apoptotic neutrophils and bacteria uptake via macrophages [20, 21].CpG-ODN are manufactured easily, low-cost, and stable. They demonstrate outstanding safety profiles and do not register any adverse reactions in the case of administration [22].

The paper aims to identify the genes that contribute to the healing of skin wounds, which express differentially in injured skin in diabetic dogs in response to different treatments. Moreover, we aim to select the molecules potentially beneficial for treating injured skin in diabetic dogs and the molecular mechanisms that control skin regeneration.

## Methods

### Experimental animals

Commercially from Al-Fahad Trading Company for Animals (Abu-Rawash, Giza, Egypt), thirty-six intact local breed mongrel canines of both sexes, aged 2–4 years, and weighing 12–15 kg were acquired. The animals were maintained in accordance with established environmental hygiene, nutritional, and medical standards. The research was conducted with the approval of the institutional animal ethics committee at the Faculty of Veterinary Medicine, Benha University (BUFVTM 19-01-23). Additionally, all procedures were implemented in accordance with pertinent guidelines and regulations regarding animal welfare. The investigation is noted in line with the ARRIVE guidelines. The diabetogenic action was achieved through one intravenous injection of 30 mg/kg bwt [23] streptozotocin (STZ) were obtained from Sigma Aldrich, USA.

### Preparing PRP & PRF by double centrifugation

For each dog, a blood sample was taken from a jugular vein. One blood sample was put into vacuum tubes of blood sampling with a solution of citrate-phosphate-dextrose to prepare PRP. Another blood sample was collected without anticoagulant in a dry glass tube for PRF preparation [24] (approximately 1 ml from PRP & PRF were applied).

### CpG-ODN phosphorothioated ODN D-SL03

In the lyophilized solid form, this material was produced by Trilink Biotechnologies Company, San Diego, California, the United States of America, with the sequence 5’ TCG CGA ACG TTC GCC GCG TTC GAA CGC GG 3’. To achieve the required dosage, 75 ug CpG-ODN/dog was provided by the subcutaneous injection [25].

### Study groups

Thirty six dogs were distributed randomly to 6 equivalent groups. All members of the groups were subjected to surgical induction of the full thickness 3 cm incised wound at the midline between two scapulas (wither area). The operation was carried out by injectable general anesthesia by administration of IM xylazine Hcl 2.0 mg/ kg (Xylaject^®^, Adwia, Egypt). Anesthesia was induced with IV propofol [26] (Diprivan 10, Aspen Pharma.Dublin.Ireland).

The treatment protocols of the groups were organized as follows:

- **Group 0** (control group): not receive any treatment.
- **Group 1** (CpG-ODN treatment): receive 75 ug CpG-ODN/dog was injected subcutaneously daily.
- **Group 2 (**PRP treatment): liquid PRP applied to the wounds daily.
- **Group 3** (PRP & CpG-ODN treatment): liquid PRP applied to the wounds daily plus receiving 75 ug CpG-ODN/dog daily.
- **Group 4 (**PRF treatment): PRF applied to the wounds daily.
- **Group 5** (PRF & CpG-ODN treatment): PRF applied to the wounds daily plus receiving 75 ug CpG-ODN/dog.

### Evaluation of wound healing

The groups were clinically evaluated on 0, 1st, 2nd and 3rd weeks following the surgical induction using digital photos of the wounds with the ruler to measure the wound size in mm and wound contraction rates (%), which were computed to control and treated wounds using these equations to:

$$WHR\% {\rm{ }} = \left( {iWA - {\rm{ }}fWA} \right)\,\,/\,iWA{\rm{ }} \times {\rm{ }}100$$

WHR is the wound healing rate, iWA is the initial wound area and fWA is the final wound area [27].

#### Histopathological evaluation of wound healing

A histopathological examination was conducted on skin wound specimens that were obtained three weeks following the surgery. We fixed the materials in 10% neutral buffered formalin. The skin tissues were progressively dehydrated and embedded in paraffin after complete fixation. Using Hematoxylin and eosin (H&E) for routine light microscopic examination and Masson’s trichrome stain to identify connective tissue maturation, five-µm-thick microscopic sections were prepared and stained in accordance with Bancroft and his colleagues [28].

The scoring system of Karayannopoulou and his colleagues [29] was employed to evaluate the degree of collagen production, re-epithelialization process, neovascularization, and cellular infiltration in five sections. Sections were assigned the following scores in relation to collagen production: 0 was classified as normal, 1 as a mild increase, 2 as a mild to moderate increase, 3 as a moderate increase, 4 as a moderate to marked rise, 5 as a marked rise, and 6 as an extensive marked increase. The degree of cellular infiltration was assessed by calculating the sum of inflammatory cells detected per high power field (HPF) (X400). The score was assigned as follows: 0 = ˂ 3 inflammatory cells, 1 = 3 to 10 inflammatory cells, 2 = 11 to 20 inflammatory cells, 3 = 21 to 30 inflammatory cells, 4 = 31 to 40 inflammatory cells, and 5 = ≥ 41 inflammatory cells. The angiogenesis score was estimated in five HPFs by estimating the number of blood vessels and capillary buds as follows: 1 represents 3 to 10 new vessels, 2 represents 11 to 30 new vessels, and 3 represents ≥ 31 new vessels found per HPF. The epithelium’s thickness over the lesion was scored as follows: 0 (thickness similar to that of normal epithelium), 1 (slightly increased thickness), 2 (moderately increased thickness), and 3 (markedly increased thickness).

### Molecular evaluation (Assessment of wound healing genes expression using PCR-Array)

#### Tissue handling

The wounds that had contracted were harvested and deposited in cryo tubes. The tubes were then stored in RNAlater solution (Qiagen-GmbH Hilden, Germany) at -80 °C, with 10 µL of the solution per 1 mg of tissue. The monofilamentous nonabsorbable suture material was used to suture the resultant wounds in a simple interrupted manner to ensure healing by first intention. The wounds were then stored for further study.

### Total RNA extraction

The total RNA extraction was performed in accordance with the manufacturing instructions using the Easy Red TM reagent from Intron Biotechnology (Korea). The rotor Tissue Ruptor (Qiagen, GmbH, Germany) was used to homogenize nearly 100 mg of tissue in a microcentrifuge tube containing 750 µL of Trizol solution. In order to synthesize cDNA, ten µL of RNA (2 µg) from each sample of the same cohort was collected.

### RNA spectrophotometric quantification

The RNA purity and concentration were determined by calculating the absorbance in a Spectrostarnano (BMG Lab Tec, GmbH, Germany). The A260/A280 rate of 1.8-2.0 was observed in pure RNA.

### cDNA synthesis

The RT2First Strand Kit (Qiagen, GmbH) was employed to synthesize cDNA. Approximately 5 µg of RNA was employed in conjunction with a genomic DNA elimination mixture, as per the manufacturing instructions. For each sample in the reverse transcription mixture, ten µL of this mixture was utilized.

### Real-Time PCR for RT2 profiler PCR array

The real-time thermocycler PCR (Applied Biosystem 7500 Fast Real-time PCR, USA) was employed to mix approximately 25 µL of RT² Profiler PCR Array: Dog Wound Healing (Qiagen-GmbH Cat. no. PAFD-121Z, Germany) and RT² SYBR^®^ Green qPCRMastermix (Qiagen-GmbH, Germany) PCR components. The real-time cycler was programmed to operate at 95 °C for 10 min, followed by 40 cycles of 95 °C for 15 s and a final period of 60 °C for one minute. The Central Laboratory, Faculty of Veterinary Medicine, Benha University, was the site of all genetic evaluations.

Healing of injuries involve three phases, the RT² Profiler PCR Array contained 84 genes that were essential in each phase. These genes included extracellular matrix (ECM) remodeling factors, inflammatory cytokines and chemokines, growth factors, and primary signaling molecules. This array can be employed to analyze the expression of the focused panel of genes in tissue injury, wound healing, and repair via real-time PCR.

### Data analysis and statistics

We exported the CT values to the Excel file to generate a table and uploaded it to the data analysis website at http://www.qiagen.com/geneglobe. We distributed the samples into control and test groups. Raw data were normalized dependent on mean of (ACTB) Actin beta, (GAPDH) Glyceraldehyde-3-phosphate dehydrogenase, (B2M) Beta-2-microglobulin, (HPRT1) Hypoxanthine phosphoribosyltransferase 1 and (RPLP1) Ribosomal protein, large, P1 as a housekeeping genes. The website calculated fold change/regulation. Furthermore, fold change was calculated employing the 2^-(∆∆Ct) formula [30]. The fold-change results are biologically meaningfully demonstrated by fold-regulation. The fold-change values exceeding 1 indicate a positive or up-regulation, and the fold-regulation is equivalent to the fold-change. Fold-change values that are less than 1 indicate a negative or down-regulation, and the fold-regulation is the negative inverse of the fold-change. The p values were determined by conducting a Student’s t-test on the replicate 2^-(∆∆Ct) values for each gene in the control and treatment groups. p values that were less than 0.05 were indicated in red. Additionally, the website displays a heat map, cluster graph, and volcano plot. The data analysis report was exported from the QIAGEN web portal at Gene Globe. The other collected data were statistically analyzed utilizing the One-Way ANOVA with Duncan’s multiple range tests [31].

## Results

### Clinical evaluation of wound healing

**Fig. 1:**
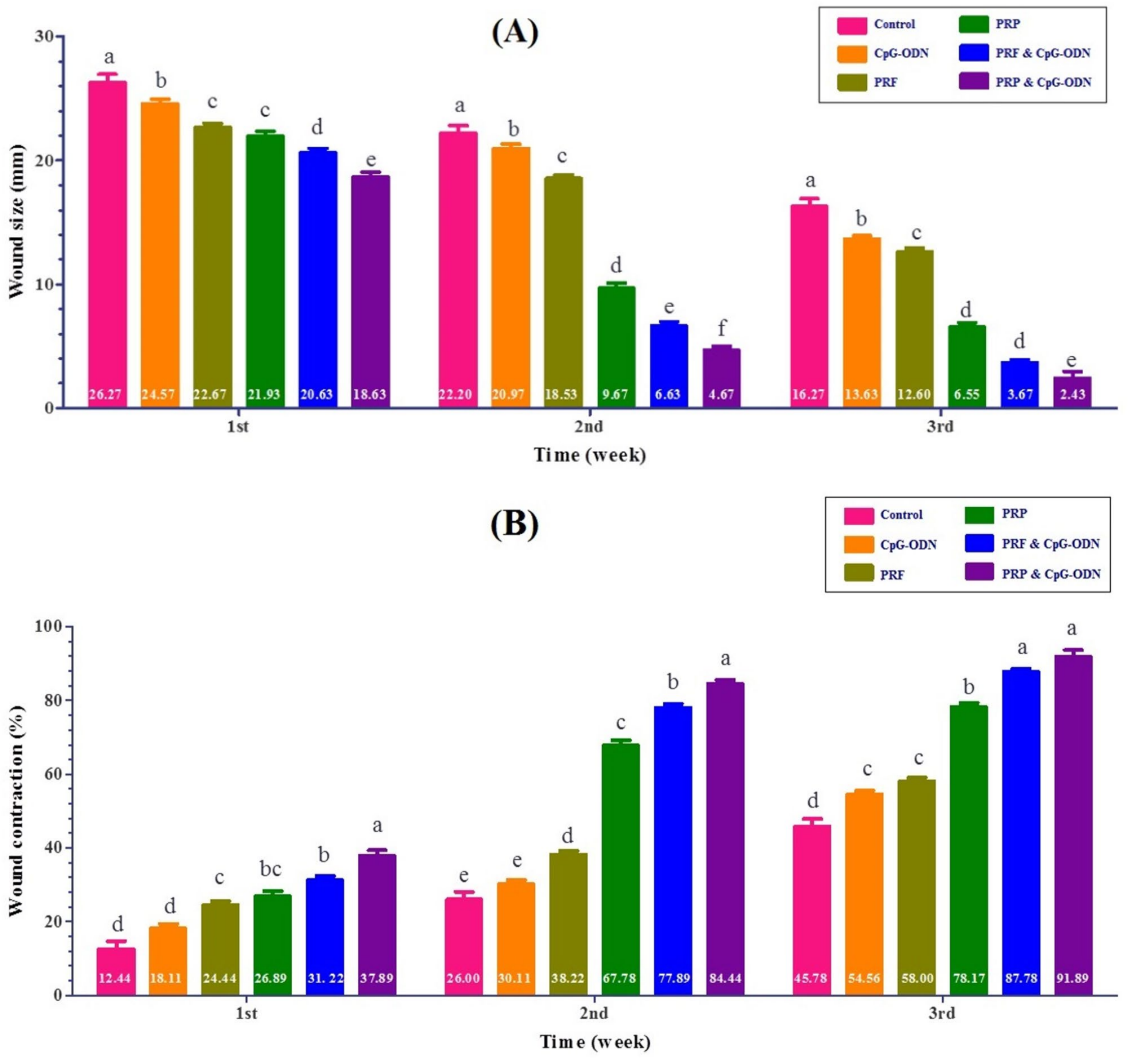
The diagram showing wound healing changes in dog between control and different treatment groups over 3 weeks (*P* < 0.05): (**A**) Wound size (mm). (**B**) Wound contraction (%). Columns with different letter at same time point were significantly diverse at P<0.05

Wound size & contraction rates (%) as an indicator for wound healing was estimated, where significant higher wound size (*P* < 0.05) were found at 1st week post-surgery and gradually decreased in subsequent weeks in control, CpG-ODN, PRF, PRP, PRF & CpG-ODN and PRP& CpG-ODN groups, respectively (Fig. 1A). Meanwhile, wound contraction % were significantly (*P* < 0.05) greater in the PRP& CpG-ODN, PRF & CpG-ODN, PRP, PRF, CpG-ODN, and control groups, respectively (Fig. 1B**)** at 3rd week post-surgery.

### Histopathological evaluation

Histopathological evaluation of the parameters in the healing of wounds helped detect some differences between the control group and treated ones (Table 1), overall favoring the group treated with PRP & CpG-ODN.

**Fig. 2:**
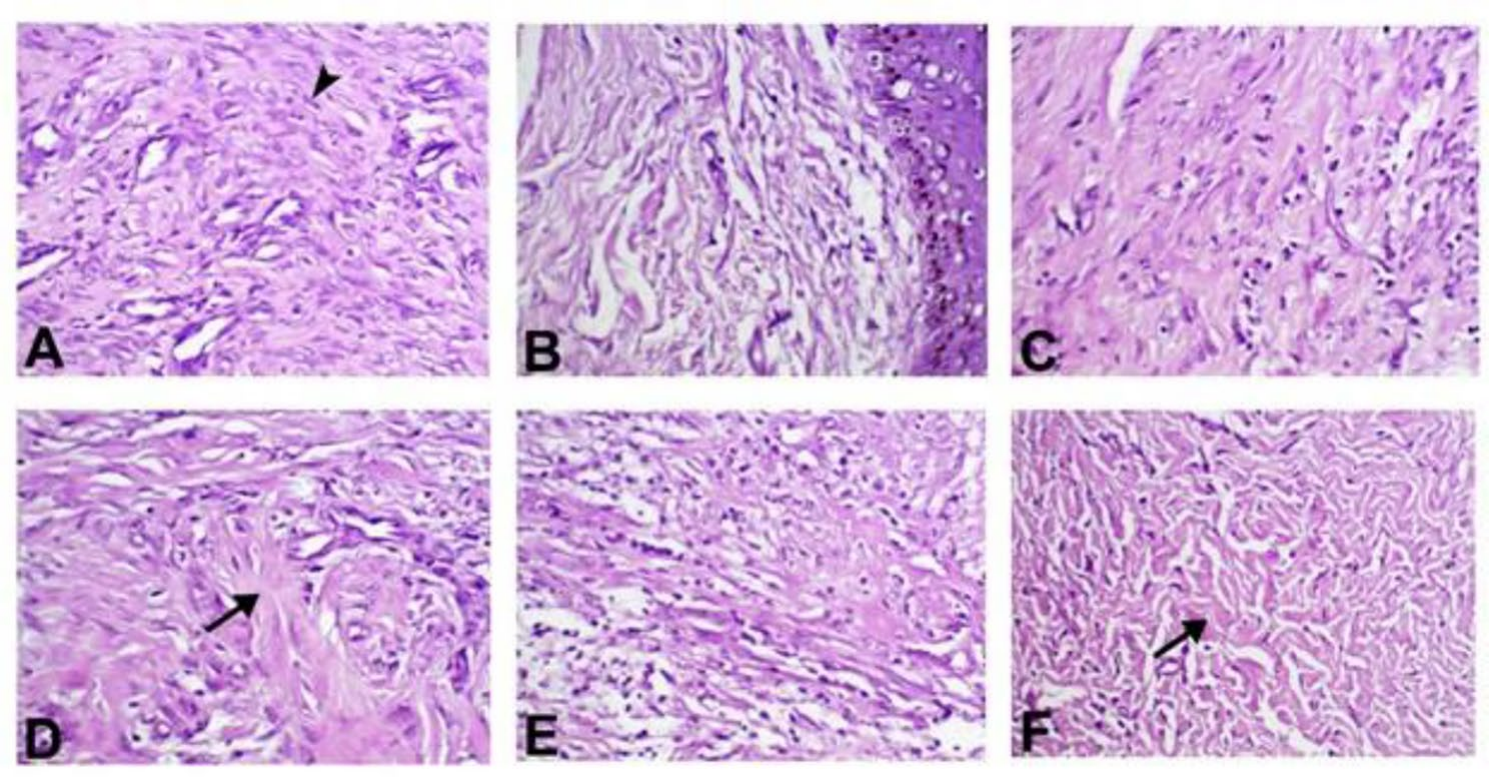
Photomicrograph showing collagen production in dermis of skin wound healing in dog on 3rd week post-surgery (X20, H&E stain). (**A**) Control group revealing granulation tissue formation with increased fibroblasts (arrowhead) and few collagen. Collagen deposition was increased in different treatment groups (**B**) CpG-ODN group; (**C**) PRP group; (**D**) PRP & CpG-ODN group; (**E**) PRF group and (**F**) PRF & CpG-ODN group. Marked increase in collagen production (arrow) was observed in groups PRP & CpG-ODN and PRF & CpG-ODN respectively

**Fig. 3:**
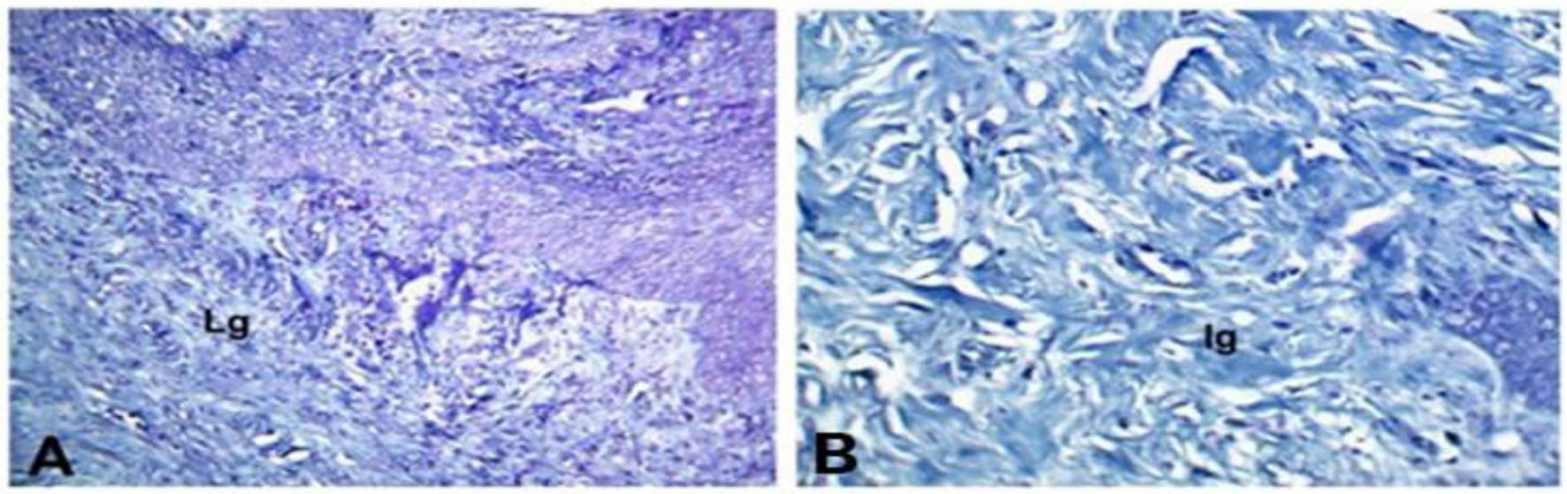
Figure symbolizes Masson’s Trichrome Staining of collagen deposition in skin wound healing in dog on 3rd week post-surgery (X20). (**A**) The control group is characterized by a light green (Lg) color. (**B**) The intense green (Ig) color of the PRP & CpG-ODN group indicates that the collagen fibers are highly mature

The collagen production score, as illustrated in Table 1, dramatically raised (P < 0.05) in the PRP & CpG-ODN and PRF & CpG-ODN groups compared with control and other treated groups (Fig. 2). The green color of the collagen deposition demonstrated by Masson’s trichrome staining was the most intense in the PRP & CpG-ODN group, suggesting highly mature collagen fibers (Fig. 3).

**Table 1:** Histopathological evaluation of wound healing on 3rd week post-surgery

Groups Parameter	Group0: Control	Group1: CpG-ODN	Group2: PRP	Group3: PRP & CpG-ODN	Group4: PRF	Group5: PRF & CpG-ODN
Collagen production	1 ± 0.32^c^	1.4 ± 0.24^c^	2.6 ± 0.24^b^	4.4 ± 0.20^a^	2.2 ± 0.20^b^	3.8 ± 0.24^a^
Cellular infiltration	2.4 ± 0.24^b^	2 ± 0.16^b^	3.8 ± 0.37^a^	2.4 ± 0.24^b^	3.6 ± 0.5^a^	2.6 ± 0.24^b^
Neovascularization	2.2 ± 0.20^a^	2 ± 0.32^a^	2 ± 0.32^a^	1.8 ± 0.20^a^	2.6 ± 0.24^a^	2.2 ± 0.20^a^
Re-epithelialization	0.8 ± 0.37^c^	1.4 ± 0.24^bc^	2 ± 0.32^ab^	2.6 ± 0.24^a^	1.8 ± 0.20^ab^	2.2 ± 0.20^ab^

Data is expressed as mean ± standard error. Different letters mean values in the same row are statistically significant (P < 0.05)

**Fig. 4:**
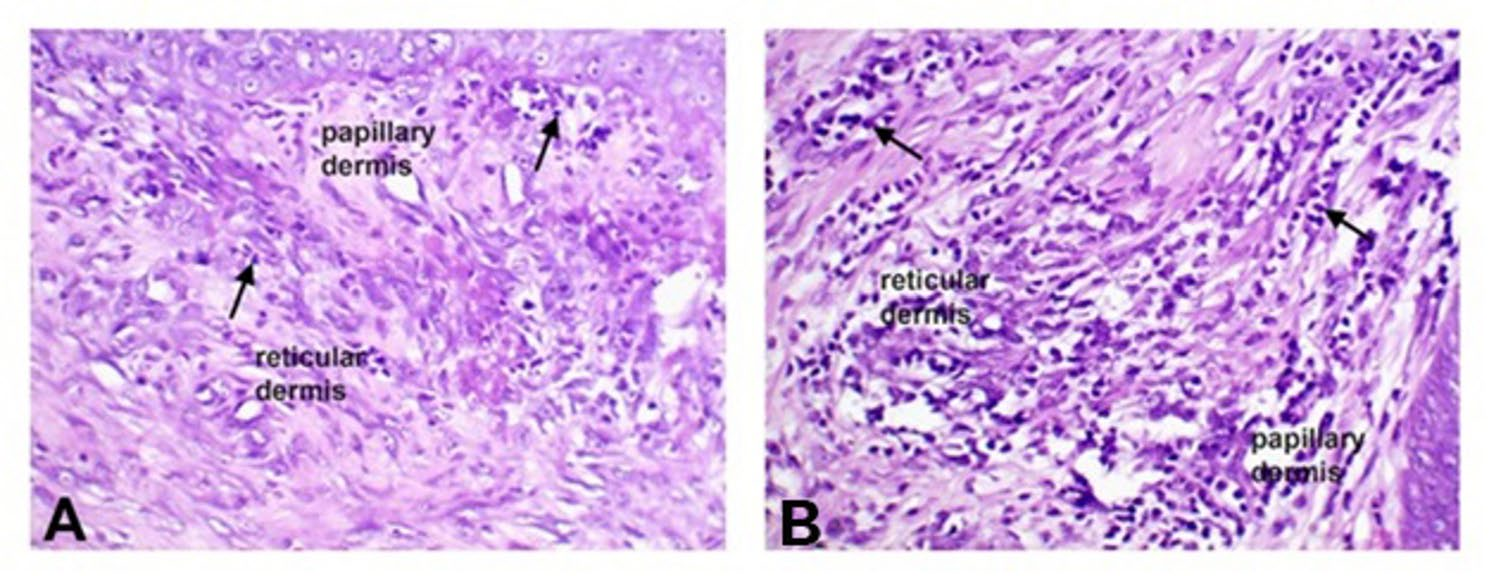
This figure illustrates the inflammatory cellular infiltration in the dermis of a skin lesion healing in a dog on the third week following surgery (X20, arrow, H&E stain).**(A)** The control group exhibited a minimal number of inflammatory cells in both the papillary and reticular dermis. **(B)** The PRP group exhibits a high number of inflammatory cells in both the papillary and reticular dermis.

The cellular infiltration score ranged from 2 to 3.8 in all groups. Higher numbers of inflammatory cells were found in the PRP and PRF groups, but only some inflammatory cells were detected in the CpG-ODN group, then the other groups of the control and PRP & CpG-ODN (Fig. 4). There were significant differences (P < 0.05) in the PRP and PRF groups compared to the other groups (Table 1).

The mean number of new vessels detected per HPF on the third week post-surgery was higher in the lesions treated with PRF. Nevertheless, there was no significant difference between the control and other treated groups and PRF group (Table 1).

**Fig. 5:**
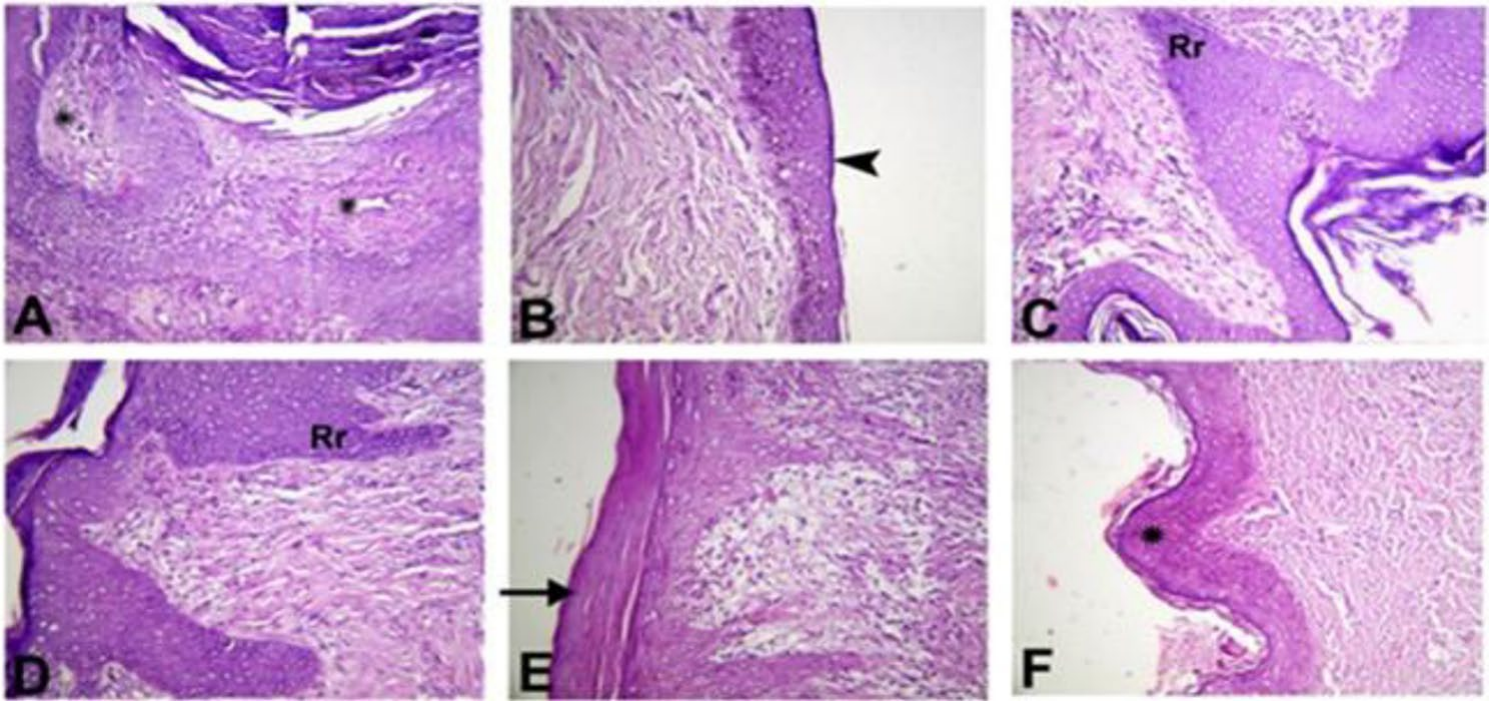
Photomicrograph represents re-epithelialization of skin wound healing in dog on 3rd week post-surgery (X20; H&E stain). (**A**) Control group showing poor keratinocyte migration with incomplete epithelization (asterisk). (**B**) CpG-ODN group showing incomplete formation of the keratin layer (arrowhead). (**C**,** D**) PRP; PRP & CpG-ODN groups showing complete epithelialization with rete ridges “Rr” formation. (**E**) PRF group revealing complete epithelialization with incomplete differentiation of the cornified layer characterized by parakeratosis (arrow). (**F**) PRF & CpG-ODN group showing complete epithelialization represented by asterisk

Re-epithelialization occurred in most groups. However, there were dramatic differences (P < 0.05) in the control group in comparison to others (Table 1). Multifocal areas of incomplete epithelization with poor keratinocyte migration were occasionally observed in the control group (Fig. 5A). The PRP and PRP & CpG-ODN groups illustrated complete epithelialization with rete ridges formation in essential granulation tissue (Figs. 5C & D). Moreover, incomplete formation or differentiation of the keratin layer was seen in the CpG-ODN or PRF group (Figs. 5B& E).

### Molecular evaluation

**Fig. 6:**
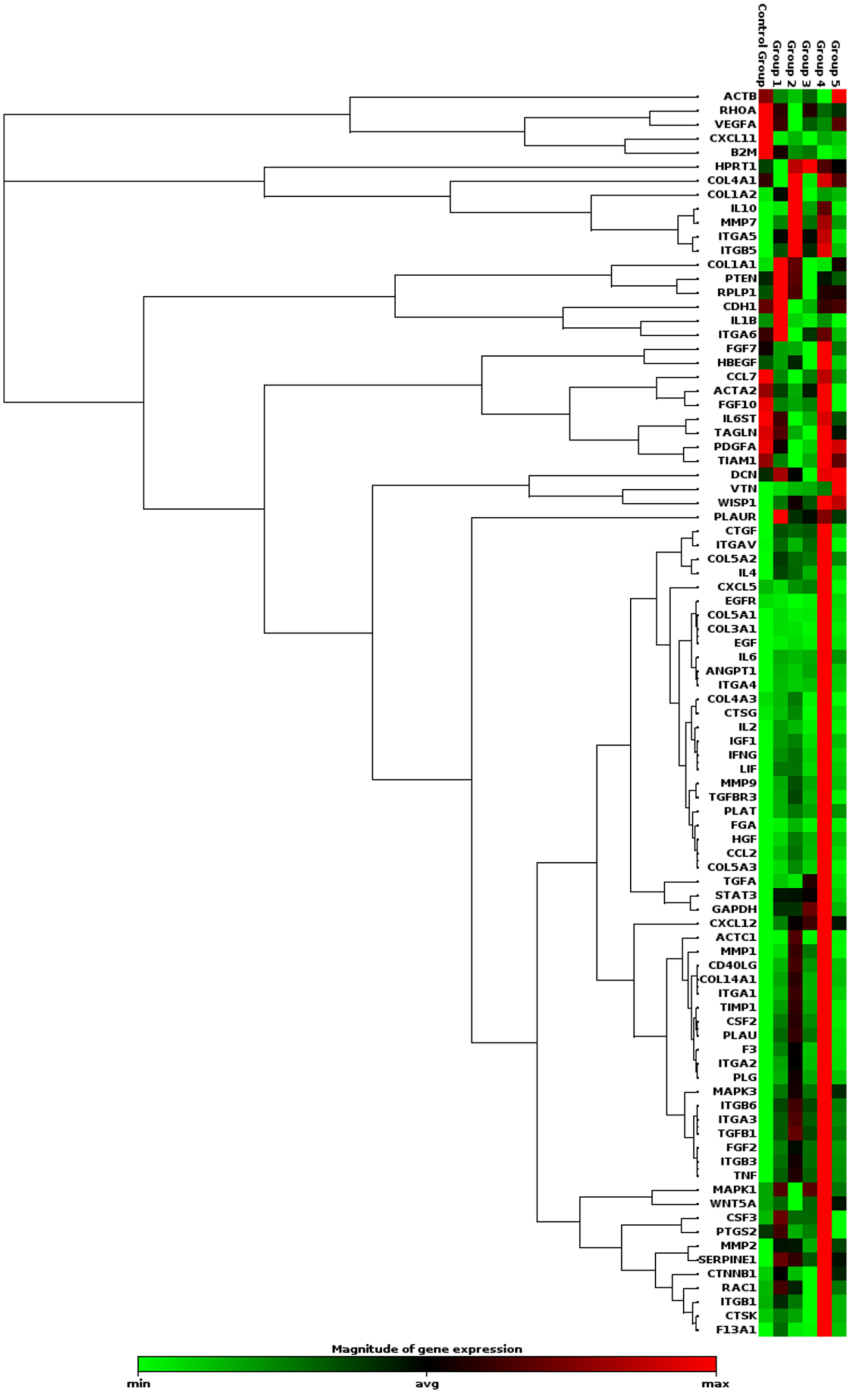
Diagram showing clustergram of all groups represents non-supervised hierarchical clustering of all wound healing genes displaying a heat map with dendrograms. Rows represent genes & columns represent different treatment groups; color scale indicates relatives gene expression level in each gene “red = up regulation” passing to “black = no change” and “green = down-regulation”

To explain the noted differences in the genes of wounds in response to different treatments among groups. We utilized pathway-focused RT-PCR arrays (Qiagen, Cat. no. PAFD-121Z) to analyze the expression profile of full-thickness excisional wounds three weeks post-wounding. These arrays contained 84 genes that were intended to be included in wound healing. The cluster graph (Fig. 6; Table 2) summarizes the gene expression profiles that were analyzed.

**Table 2:** Fold regulation and p values of the wound healing genes expression in treated groups compared to control one

Gene Symbol	Fold Regulation (comparing to control group)									
	Group 1		Group 2		Group 3		Group 4		Group 5	
	Fold Regulation	*p*-Value	Fold Regulation	*p*-Value	Fold Regulation	*p*-Value	Fold Regulation	*p*-Value	Fold Regulation	*p*-Value
ACTA2	-1.26	0.08385	-1.44	0.13597	-1.2	0.52039	1.1	0.52122	-1.62	0.05909
ACTC1	1.64	0.07841	40.6	0.00111	2.12	0.20321	61.56	1.7E-05	2.32	0.0175
ANGPT1	5.95	0.00007	4.62	0.00704	7.07	0.00162	36.91	0.00045	4.7	0.00282
CCL2	2.71	0.01708	4.93	0.00394	2.84	0.00711	15.09	0.00156	2.03	0.01923
CCL7	-3.56	0.00039	-15.96	1.1E-05	-3.33	0.00025	-1.14	0.13781	-4.04	3.9E-05
CD40LG	4.98	0.00319	19.05	0.00062	6.74	2.6E-05	29.64	0.00062	4.26	0.00438
CDH1	1.21	0.15182	-1.93	0.00213	-1.61	0.03259	-1.07	0.54484	-1.04	0.76411
COL14A1	6.26	0.02359	18.78	0.00049	5.61	0.00796	31.47	0.00752	4.81	0.01735
COL1A1	3.17	0.02351	2.45	0.00804	-1.17	0.4855	-1.03	0.77069	2.1	0.01647
COL1A2	1.75	0.04488	2.65	0.00613	-1.08	0.72162	1.25	0.18585	1.15	0.47402
COL3A1	2.95	0.01732	2.78	0.01788	1.4	0.22354	33.78	0.00509	2.09	0.01856
COL4A1	-1.42	0.09764	1.2	0.2273	-1.38	0.17315	1.16	0.30965	1.03	0.8943
COL4A3	1.45	0.05186	2.31	0.00753	-1.4	0.10252	7.16	0.00047	-1.67	0.05608
COL5A1	5.13	0.00007	2.89	0.02136	3.46	0.00252	63.33	9.9E-05	4.43	0.00197
COL5A2	1.52	0.17148	1.4	0.24197	1.35	0.19811	2.34	0.02397	1.31	0.26395
COL5A3	1.42	0.28671	2.35	0.0035	1.54	0.04323	7	0.00717	1.07	0.76605
CSF2	3.18	0.02861	5.86	0.00283	2.9	0.00183	9.47	0.0002	1.26	0.31601
CSF3	2.68	0.04065	1.47	0.18719	1.48	0.23085	3.56	0.00069	-1.68	0.11975
CTGF	5.88	0.00168	5.26	0.00229	5.7	0.01048	15.18	0.00358	2.5	0.02009
CTNNB1	2.16	0.0081	1.1	0.76075	-1.36	0.20677	3.55	0.00046	1.95	0.05433
CTSG	1.56	0.09189	2.31	0.00628	-1.39	0.1652	7.66	3.6E-05	1.18	0.55349
CTSK	1.55	0.09229	1.27	0.37094	-1.51	0.12571	4.1	0.0147	1.15	0.55631
CXCL11	-4.16	0.00478	-3.1	0.00827	-4.71	0.00415	-2.91	0.69104	-3.38	0.00707
CXCL12	2.72	0.01051	4.67	0.01125	5.44	0.00711	8.17	0.00022	4.26	0.00418
CXCL5	-1.27	0.37992	1.22	0.32254	1.34	0.29967	3.68	0.00531	-1.74	0.04164
DCN	1.64	0.02088	1.12	0.54863	-2.86	0.00633	1.85	0.00291	1.92	0.0036
EGF	3.13	0.0097	4.95	0.00873	2.92	0.00538	62.37	0.00509	4.41	0.00439
EGFR	-1.16	0.50269	-1.74	0.05794	-1.39	0.16462	7.49	2.6E-05	1.09	0.64018
F13A1	10.76	0.00108	2.27	0.02908	1.44	0.16656	34.97	0.00057	4.96	0.00767
F3	1.69	0.10376	2.45	0.0417	1.32	0.24585	3.84	0.02288	1.16	0.50618
FGA	1.52	0.14625	4.66	0.00242	1.58	0.01922	26.35	0.00092	1.8	0.0393
FGF10	-2.8	0.00658	-3.42	0.00357	-2.84	0.00729	1.03	0.82383	-7.49	0.00179
FGF2	3.2	0.03564	5.13	0.01393	3.43	0.00049	9.64	0.00105	2.68	0.00973
FGF7	-2.7	0.01381	-2.98	0.01432	-29.74	0.00312	1.85	0.06174	-1.92	0.03402
HBEGF	-1.36	0.2243	1.18	0.57303	-2.52	0.03432	2.22	0.00747	-1.75	0.11563
HGF	6.56	0.00844	19.09	0.00039	10.97	0.00943	70.5	0.00078	8.81	0.00065
IFNG	24.53	0.00077	31.89	0.00271	11.01	1.6E-05	111.28	0.00254	8.59	0.00217
IGF1	15.23	0.00064	19.5	0.0032	5.54	0.00179	76.41	0.00052	10.32	0.00244
IL10	2.85	0.00061	41.73	0.00028	8.22	0.00018	28.85	0.00612	2.03	0.03378
IL1B	2.8	0.01071	-1.42	0.16243	-1.76	0.07885	-1.02	0.84006	-1.98	0.07237
IL2	12.2	1.4E-05	9.29	0.00027	2.97	0.00057	60.4	0.00753	2.36	0.00758
IL4	107.38	0.00095	88.62	0.00028	45.26	0.00018	292.89	0	18.18	0.00643
IL6	3.15	0.00896	2.78	0.02344	3.11	0.02135	14.4	0.00409	3.78	0.00186
IL6ST	-1.35	0.26771	-3.07	0.01645	-2.35	0.02321	-1.05	0.70676	-1.8	0.07336
ITGA1	3.22	0.00119	10.55	0.00202	3.34	0.00098	16.71	0.0003	2.43	0.02694
ITGA2	1.54	0.14645	2.5	0.00911	1.39	0.29181	4.04	0.00365	1.18	0.54813
ITGA3	5.78	0.01165	10.75	0.00027	5.76	3.1E-05	15.92	0.00021	4.39	0.00697
ITGA4	2.89	0.01233	2.41	0.00658	2.78	0.00151	15.82	5.3E-05	2.37	0.0133
ITGA5	1.6	0.0395	2.23	0.049	1.59	0.01665	2.09	0.00907	1.05	0.83147
ITGA6	1.26	0.26672	-1.67	0.07077	-1.16	0.35939	1.06	0.80356	-1.46	0.14104
ITGAV	1.27	0.19648	1.13	0.56775	1.27	0.35904	1.94	0.0055	-1.01	0.91777
ITGB1	1.43	0.28377	1.18	0.47328	-1.31	0.22668	2.34	0.02169	-1.01	0.90123
ITGB3	5.65	0.00228	9.93	8.2E-05	5.75	0.00052	17.66	0.00486	4.42	0.01093
ITGB5	1.46	0.19061	2.35	0.00777	1.56	0.06704	2.28	0.00516	1.16	0.57716
ITGB6	11.26	0.01096	18.85	0.00021	11.14	0.0013	29.54	2.5E-05	8.23	0.00392
LIF	9.69	0.00049	9.77	0.0057	3.3	0.00062	33.35	0.00035	3.96	0.00156
MAPK1	1.58	0.12821	-1.27	0.32007	1.6	0.1278	2	0.0121	1.12	0.51022
MAPK3	2.79	0.01292	4.64	0.00023	2.84	0.02092	7.75	0.00607	3.83	0.00069
MMP1	1.47	0.09953	5.15	0.0011	2.81	0.03683	7.86	0.00201	1.09	0.72472
MMP2	2.43	0.02362	2.41	0.02638	1.49	0.04004	4.08	0.00078	2.14	0.01941
MMP7	3.18	0.00446	10.11	0.00439	3.52	0.00113	8.47	0.00133	2.68	0.01397
MMP9	5.76	0.01007	10.99	0.00679	5.79	5.5E-05	30.13	0.00054	4.43	0.00328
PDGFA	-1.47	0.13197	-3.38	0.01844	-2.76	0.02368	1.02	0.96181	-1.04	0.71198
PLAT	6.1	0.00654	9.99	0.00038	7	0.00013	35.13	0.00898	8.21	0.00044
PLAU	3.1	0.0132	5.24	0.00245	2.9	0.00101	8.1	0.00076	1.35	0.26514
PLAUR	47.33	0.00369	19.86	0.00023	23.09	0.00127	36.12	0.00095	19.3	0.00004
PLG	11.98	0.00034	37.85	0.00022	12.68	1.5E-05	70.39	0.00259	8.79	0.00243
PTEN	1.42	0.18285	1.18	0.55876	-1.44	0.1906	1.03	0.97038	-1.08	0.65629
PTGS2	1.38	0.06154	-1.64	0.09345	-1.31	0.26238	1.98	0.09758	-2.77	0.00856
RAC1	1.62	0.16038	1.37	0.25935	-1.22	0.36685	2.08	0.03637	1.16	0.61369
RHOA	-1.43	0.22233	-3.87	0.00713	-1.47	0.1604	-2.09	0.04658	-1.79	0.0513
SERPINE1	13.23	0.00927	11.36	0.00166	6.68	0.00022	18.45	9.3E-05	9.15	0.00194
STAT3	1.44	0.19925	1.45	0.14006	1.5	0.07415	1.96	0.02244	1.08	0.7703
TAGLN	-1.31	0.29688	-3.07	0.02591	-5.41	0.01382	1.06	0.92277	-1.7	0.12197
TGFA	1.32	0.30639	1.14	0.52979	2.74	0.02818	4.06	0.01422	1.16	0.47529
TGFB1	5.79	0.00476	11.18	0.00015	6.28	0.00089	15.74	0.00012	4.77	0.00127
TGFBR3	1.62	0.02504	2.48	0.00303	1.55	0.06938	5.07	7.6E-05	1.09	0.70727
TIAM1	-1.34	0.31435	-1.58	0.05654	-1.42	0.10499	1.1	0.61708	-1.05	0.71898
TIMP1	1.61	0.03707	2.93	0.01419	1.56	0.08201	4.23	0.00712	1.09	0.74619
TNF	11.96	0.00335	20.68	0.00477	11.91	5E-06	36.14	4.9E-05	8.53	0.00063
VEGFA	-1.54	0.13624	-12.08	0.01909	-2.68	0.02934	-3.45	0.01814	-1.43	0.19269
VTN	3.06	0.00199	5.36	0.00194	6.14	0.00428	9.16	0.00091	33.25	4.9E-05
WISP1	20.17	0.00016	39.59	0.00747	24.99	0.00074	73.67	0.00801	65.06	0.00077
WNT5A	1.5	0.12183	-3.43	0.84464	1.53	0.15449	4.33	0.02322	2.18	0.01042
	-1.47	0.14708	-1.64	0.07917	-1.39	0.16794	-1.86	0.04549	1.14	0.55736
	-1.25	0.24492	-1.53	0.12219	-1.48	0.12742	-1.79	0.04694	-1.71	0.06325
	1.57	0.04128	1.58	0.10663	2.01	0.00117	2.43	0.00041	1.19	0.3429
	-1.28	0.18345	1.3	0.20967	1.38	0.07318	1.16	0.39025	1.08	0.69633
	1.5	0.1041	1.23	0.28954	-1.35	0.20267	1.17	0.45309	1.16	0.49381

### Gene expression regulation in group 1 (CpG-ODN treated group)

**Fig. 7:**
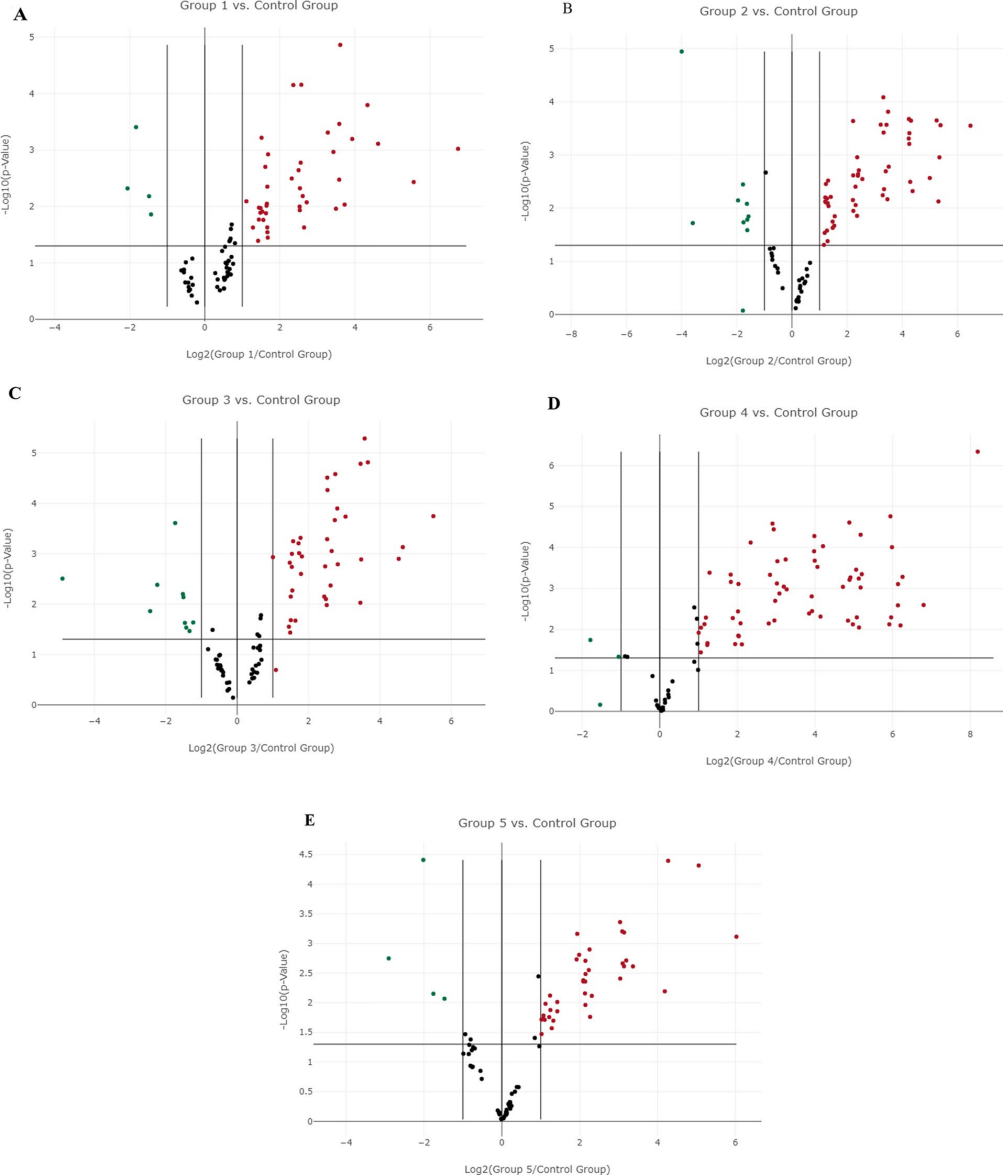
Volcano plots illustrates the significant expression alterations of wound healing genes among group 1 (**A**), group 2 (**B**), group 3 (**C**), group 4 (**D**), and group 5 (**E**) in comparison to the control group. The y-axis displays the statistical significance of the factor changes in gene expression, while the x-axis displays the log2 of these changes. The two outer vertical lines denote the selected fold regulation threshold, while the center vertical line denotes unchanged gene expression. The selected p-value threshold is denoted by the horizontal line. The selected fold regulation and p-value thresholds (*p* < 0.05) are met by genes with data points in the far upper left (down-regulated) and far upper right (up-regulated) sections

From total 84 genes there are up regulation with significance (*p* < 0.05) in 42 genes (ANGPT1, CCL2, CD40LG, COL14A1, COL1A1, COL3A1, COL5A1, CSF2, CSF3, CTGF, CTNNB1, CXCL12, EGF, F13A1, FGF2, HGF, IFNG, IGF1, IL10, IL1B, IL2, IL4, IL6, ITGA1, ITGA3, ITGA4, ITGB3, ITGB6, LIF, MAPK3, MMP2,MMP7, MMP9, PLAT, PLAU, PLAUR, PLG, SERPINE1, TGFB1, TNF, VTN, WISP1) and significant down regulation in 4 genes (CCL7, CXCL11, FGF10, FGF7) (Table 2; Fig. 7A).

### Gene expression regulation in group 2 (PRP treated group)

From total 84 genes there are up regulation with significance (*p* < 0.05) in 52 genes (ACTC1, ANGPT1, CCL2, CD40LG, COL14A1, COL1A1, COL1A2, COL3A1, COL4A3, COL5A1, COL5A3, CSF2, CTGF, CTSG, CXCL12, EGF, F13A1, F3, FGA, FGF2, HGF, IFNG, IGF1, IL10, IL2, IL4, IL6, ITGA1, ITGA2, ITGA3, ITGA4, ITGA5, ITGB3, ITGB5, ITGB6, LIF, MAPK3, MMP1, MMP2, MMP7, MMP9, PLAT, PLAU, PLAUR, PLG, SERPINE1, TGFB1, TGFBR3, TIMP1, TNF, VTN, WISP1) and significant down regulation in 9 genes (CCL7, CXCL11, FGF10, FGF7, IL6ST, PDGFA, RHOA, TAGLN, VEGFA ) (Table 2; Fig. 7B).

### Gene expression regulation in group 3 (PRP &CpG-ODN treated group)

From total 84 genes there are up regulation with significance (*p* < 0.05) in 37 genes ( ANGPT1, CCL2, CD40LG, COL14A1, COL5A1, CSF2, CTGF, CXCL12, EGF, FGF2, HGF, IFNG, IGF1, IL10, IL2, IL4, IL6, ITGA1, ITGA3, ITGA4, ITGB3, ITGB6, LIF, MAPK3, MMP1, MMP7, MMP9, PLAT, PLAU, PLAUR, PLG, SERPINE1, TGFA, TGFB1, TNF, VTN, WISP1) and significant down regulation in 10 genes (CCL7, CXCL11, DCN, FGF10, FGF7, HBEGF, IL6ST, PDGFA, TAGLN, VEGFA) (Table 2; Fig. 7C).

### Gene expression regulation in group 4 (PRF treated group)

From total 84 genes there are up regulation with significance (*p* < 0.05) in 62 genes (ACTC1, ANGPT1, CCL2, CD40LG, COL14A1, COL3A1, COL4A3, COL5A1, COL5A2, COL5A3, CSF2, CSF3, CTGF, CTNNB1, CTSG, CTSK, CXCL12, CXCL5, EGF, EGFR, F13A1, F3, FGA, FGF2, HBEGF, HGF, IFNG, IGF1, IL10, IL2, IL4, IL6, ITGA1, ITGA2, ITGA3, ITGA4, ITGA5, ITGB1, ITGB3, ITGB5, ITGB6, LIF, MAPK1, MAPK3, MMP1, MMP2, MMP7, MMP9, PLAT, PLAU, PLAUR, PLG, RAC1, SERPINE1, TGFA, TGFB1, TGFBR3, TIMP1, TNF, VTN, WISP1, WNT5A) and significant down regulation in 2 genes ( RHOA, VEGFA) (Table 2; Fig. 7D).

### Gene expression regulation in group 5 (PRF &CpG-ODN treated group)

From total 84 genes there are up regulation with significance (*p* < 0.05) in 39 genes (ACTC1, ANGPT1, CCL2, CD40LG, COL14A1, COL1A1, COL3A1, COL5A1, CTGF, CXCL12, EGF, F13A1, FGF2, HGF, IFNG, IGF1, IL10, IL2, IL4, IL6, ITGA1, ITGA3, ITGA4, ITGB3, ITGB6, LIF, MAPK3, MMP2, MMP7, MMP9, PLAT, PLAUR, PLG, SERPINE1, TGFB1, TNF, VTN, WISP1, WNT5A) and significant down regulation in 4 genes (CCL7, CXCL11, FGF10, PTGS2) (Table 2; Fig. 7E).

## Discussion

Regenerative medicine introduce new sights for treatments of diseased or damaged animal tissues and tissue regeneration [3]. One of such treatments is PRP which represents plasma part that lack of blood cells, when topically administrated in infected wounds with MRSA in rats. It accelerated the wound healing by decreasing the inflammation and improving proliferative phase [32]. Another fibrin matrix supports wound healing is PRF. In case of material loss tissue injuries accompanied via a multifactorial disease, PRF accelerates wound healing and tissue regeneration [33]. Our studies give another insight in regenerative medicine is to combine PRP or PRF with another material enhances its effect like CpG-ODN in healing of complicated wound. Several studies continue in the same approach. Bulut and his colleague’s support that in geriatric rats mesenchymal stem cells (MSCs) accelerates wound healing when used alone, moreover when combined with PRF the healing rate were enhanced [34].

In this study (Fig. 1) we observed that both PRP and PRF have positive effects on wound size & contraction %. Some studies discussed the role of both of them on wound healing and measurements of wound sizes provided comparable findings in the healing process, with > 80% wound contraction [35]. They have antimicrobial effects and immune regulation. Autologous leucocytes in the wound bed protect it from being contaminated by bacteria, causing longer protection [36]. Moreover, we found that PRP showed a better healing profile than PRF, as confirmed by our previous study [24], that the PRP and PRF had better regeneration capacity and enhanced the healing of wounds, PRP surpassed PRF. PRF is a 3D scaffold, while PRP is fluid in its consistency and can be injected directly into the host. Thus, PRP is more favorable than PRF [11].

Meanwhile, we noticed that PRF significantly increased most genes, like collagen genes and growth factors, with a significant increase in inflammatory genes and cytokines, especially IL 4 & IFNG, than PRP. This finding was compatible with findings that the skin fibroblasts’ migration exceeded 350% in PRF. Both PRP and PRF enhanced the mRNA levels of PDGF significantly, while the levels of collagen 1, TGF-beta, and fibronectin mRNA were considerably high in the PRF group [37]. In contrast, Fan and his colleagues [38] illustrated that the excess levels of cytokines and chemokines led to scar tissue formation and fibrosis at wound sites “cytokine storm”. But, in our results, the addition of CpG-ODN to both PRP and PRF treatments ameliorated this effect, reducing cytokine storm and modulating expression of almost genes under investigation as the inflammation degree and angiogenesis were deemed important for inducing skin repair. Their resolution was vital, and increased cellularity was associated with decreased biomechanical strength, including leukocyte infiltration, reduced cellularity, and vessel density, indicating a quicker and more suitable maturation and remodeling compared to the control. This finding agreed with Brancato & Albina [39].

Histopathological findings regarding PRP-treated wounds, Figs. 4 and 5 illustrated significant improvement in the healing of wounds, increases number of inflammatory cell with complete epithelialization. Evaluation after PRP revealed better cellularity, higher vascularity, more granulation tissues, and higher numbers of skin appendages resulting in better cutaneous regeneration and accelerated healing [40]. Conversely, another study found significant expression in wound healing genes markers, including dermal matrix deposition (COL 1Α1), proangiogenic (VEGF), cell migration (BFGF), as well as cell proliferation (PCNA) in response to PRP treatment in rats [41]. PRP also induced an increase in expressing catabolic marker genes, matrix metalloproteinase-1 (MMP-1), MMP-13, interleukin-6 (IL-6), TNF-α, prostaglandin E2 (PGE2), and collagen types I and III [42].

Clinically, highly significant wound contraction % was determined in the PRP & CpG-ODN followed by PRF & CpG-ODN, PRP, PRF, CpG-ODN, and control groups (Fig. 1B). Additionally, these conclusions are corroborated by our histopathological assessment which revealed higher collagen with highly intense green color by Masson’s Trichrome stain in this group (Figs. 2 and 3). These results and the expression of wound healing genes (Table 2; Fig. 6) came parallel to that reported by Chamorro and his colleagues [43], who explained that the gene expression levels of collagen 4 and 5 upregulated during the first week of healing. There was a significant gradual decrease in the inflammatory response within 2 weeks, but it disappeared after 4 weeks. They added that the tissue inhibitor of metalloproteinase-1 (TIMP1) increased quickly and, after 2 weeks, returned to control levels. Moreover, collagens and matrix remodeling enzymes demonstrated a decrease in both collagens I and III during the first 6 h, then increased at (8–28 days).

Molecular mechanism of wound healing in response to different treatments in diabetic dogs using the new molecular technique RT2 Profiler PCR Array (Qiagen) enabled us to view a focused panel of genes (84 genes) responsible for wound healing. This result coincided with Hassmann**-**Poznańska and his colleagues [44], who found considerable changes in expressing 42 genes during different phases of healing in rats using PCR Array (Qiagen). They also observed an increase in the expression of genes that are implicated in the inflammatory response, including granulocyte, interleukin 6, and macrophage chemotactic proteins, on the second day. In addition, there was a modification in the expression of various genes associated with extracellular matrix components and their remodeling enzymes. The expression of the growth factor genes, which included VEGFA, IGF1, and HBEGF, was higher during the initial stages of regeneration. However, HGF expression was at its highest on the third day. Many changes in gene expression that were associated with the remodeling of the extracellular matrix emphasized the critical function of connective tissue in the healing process. More evaluations of the potential applications of the molecules involved in enhancing this process, such as HBEGF and HGF, in clinical treatments could be administered.

The healing rate improved considerably in the PRP & CpG-ODN group, Fig. 7C illustrated up regulation with significance (*p* < 0.05) in 37 genes ( ANGPT1, CCL2, CD40LG, COL14A1, COL5A1, CSF2, CTGF, CXCL12, EGF, FGF2, HGF, IFNG, IGF1, IL10, IL2, IL4, IL6, ITGA1, ITGA3, ITGA4, ITGB3, ITGB6, LIF, MAPK3, MMP1, MMP7, MMP9, PLAT, PLAU, PLAUR, PLG, SERPINE1, TGFA, TGFB1, TNF, VTN, WISP1) and significant down regulation in 10 genes (CCL7, CXCL11, DCN, FGF10, FGF7, HBEGF, IL6ST, PDGFA, TAGLN, VEGFA). This result agreed with some studies that used PCR array to illustrate changes in expressing the genes relevant to the healing of wounds; they reported increased gene expression of mediators of inflammation, including (PTGS), (IL-1b, IL-6, and IL-10), and members of the chemokine CXCL-1, CXCL-3, and CXCL-6 in the first six hours after injury. Growth factors and integrin receptors showed upregulation, including (TGF-α and beta), (FGF-2 AND FGF-7), (VEGF), (IGF-1), (HGF), and (HB-EGF) genes during the first week of healing. The integrin family contained the genes of the extracellular matrix receptors, which included (ITGA) subunits 2, 4, and 5. Furthermore, the gene members of the colony stimulation factors two and three (CSF-2 AND CSF-3) exhibited enhanced expression. However, genes of the extracellular matrix modulating enzyme metalloproteinase 9 increased earlier but decreased at (14–28 days) [14, 45].

In Table 1, histopathological findings in the CpG-ODN group reveal low number of inflammatory cell with incomplete keratin layer (Fig. 5). These findings agreed with Hergertand his colleagues [17] that illustrated that histological analysis showed lower capillary density, lower wound cellularity, and fewer infiltrating leukocytes but showed more M2 macrophages after CpG-ODN treatments, denoting better healing of wounds on day eighteen in comparison with control. Molecular analysis of TLR-9 illustrated that the expression of receptors on keratinocytes and fibroblasts enhanced the migration upon providing a higher dose of CpG-ODN and increasing their growth factor.

The considerable rise in the rate of healing ascribed to wound infiltration by macrophages was enhanced in the early stage of wound healing [14]. Macrophages secreted TGF-β that stimulated fibroblast proliferation and migration in the wounds [46]. Li noticed that CpG ODN elevated the rate of CD4 + and CD8 + T cells and increased cytokines produced in skin wounds, e.g., TNF-α, IFN‐α, IL‐1β, IFN‐γ, IL‐6, and TGF‐β [18]. CpG ODN activate the macrophages by regulating TLR9/MyD88/ NF‐κB pathway and activating the healing of wounds partly because of immune response and fibroblast regulation [45]. Another finding supported that CpG ODN dependent increased basic growth factors of fibroblasts and the migration of keratinocytes and significantly accelerated wound re-epithelialization. This occurred autonomously with other factors (e.g., IL-1a and TGF) in the wound. CpG-ODN enhanced BFGF expression at wound sites in mice during 24 h [47]. TGF levels normally increase at the early phase of wound healing, which helps recruit inflammatory cells, collagen production, angiogenesis, as well as the remodeling of wounds [48].

The upregulation of expressing pro-inflammatory genes (including IL-1 and CCL2) appeared higher in the CpG-ODN group. This result agreed with Mitchell and Olive [49], who reported that IL-1, CCL2, and IFN factors were quickly formed in wound sites, accelerated wound repair and increased migrating keratinocytes, and produced other factors linked with the repair of wounds. In contrast Low and his colleagues [50], which explained that expressing CCL2 was upregulated in the inflammatory stage of wound healing. In its lack, re-epithelialization, angiogenesis, synthesis of collagen, and wound repair were postponed considerably.

This study’s aim is to evaluate the differential wound healing genes expression in canine with diabetes using regenerative medicine such as PRP, PRF, CpG-ODN and combination between CpG-ODN with either PRP or PRF. Our clinical, histopathological, and molecular data indicated that all treatment enhance wound healing. PRP is more preferred than PRF as it is fluid directly injected. Moreover CpG-ODN combined with PRP lead to best tissue response to healing according to the low inflammatory and fibrotic response and improved regenerative capacity. So we can concluded that PRP & CpG-ODN was superior to PRF & CpG-ODN, PRP, PRF, CpG-ODN, and the control group, respectively, for enhancing wound healing in diabetic dogs provided a new and possible technique for the veterinary or clinical wound care fields.

## Electronic supplementary material

Below is the link to the electronic supplementary material.

Supplementary Material 1

## Data Availability

The datasets used and/or analyzed during the current study available from the corresponding author on reasonable request.
