# Supplementary material for: Molecular profile PCR array of regenerative therapy (PRP, PRF& CpG-ODN) in wound healing of diabetic dogs
Source: BMC Vet Res. 2025 Jul 7;21:444. doi: 10.1186/s12917-025-04892-9 (PMC12232839; doi:10.1186/s12917-025-04892-9)
Supplement: Supplementary file 1 — Supplementary Material 1 [file 12917_2025_4892_MOESM1_ESM.docx]

**Table (3): Fold regulation and adjusted p values (BH-FDR) of the wound healing genes expression in treated groups versus control one.**

P-values obtained from statistical comparisons (Student's t-test) were adjusted using the Benjamini-Hochberg (BH) procedure to control the False Discovery Rate (FDR). This adjustment accounts for the multiple comparisons made during statistical testing, reducing the chance of false positives. All genes with Final BH-FDR values less than 0.05, indicating that they are statistically significant. Therefore, we can conclude that these genes show true differential significance, and the likelihood of them being false positives is controlled at an acceptable rate (≤ 5%).

| **Symbol** | **Final BH-FDR**  **Group 1** | **Final BH-FDR**  **Group 2** | **Final BH-FDR**  **Group 3** | **Final BH-FDR**  **Group 4** | **Final BH-FDR**  **Group 5** |
| --- | --- | --- | --- | --- | --- |
| ACTA2 | 0.128 ^NS^ | 0.170 ^NS^ | 0.527 ^NS^ | 0.600 ^NS^ | 0.097 ^NS^ |
| ACTC1 | 0.122 ^NS^ | 0.005^**^ | 0.244 ^NS^ | 0.001^**^ | 0.039^*^ |
| ANGPT1 | 0.002^**^ | 0.015^*^ | 0.006^**^ | 0.002^**^ | 0.013^*^ |
| CCL2 | 0.036^*^ | 0.011^*^ | 0.018^*^ | 0.004^**^ | 0.040^*^ |
| CCL7 | 0.006^**^ | 0.001^**^ | 0.002^**^ | 0.165 ^NS^ | 0.001^**^ |
| CD40LG | 0.016^*^ | 0.003^**^ | 0.001^**^ | 0.002^**^ | 0.015^*^ |
| CDH1 | 0.190 ^NS^ | 0.009^**^ | 0.057 ^NS^ | 0.618 ^NS^ | 0.809 ^NS^ |
| COL14A1 | 0.045^*^ | 0.003^**^ | 0.019^*^ | 0.012^*^ | 0.039^*^ |
| COL1A1 | 0.045^*^ | 0.015^*^ | 0.497 ^NS^ | 0.830 ^NS^ | 0.038^*^ |
| COL1A2 | 0.074 ^NS^ | 0.015^*^ | 0.722 ^NS^ | 0.220 ^NS^ | 0.634 ^NS^ |
| COL3A1 | 0.036^*^ | 0.028^*^ | 0.261 ^NS^ | 0.010^*^ | 0.040^*^ |
| COL4A1 | 0.141 ^NS^ | 0.269 ^NS^ | 0.217 ^NS^ | 0.361 ^NS^ | 0.912 ^NS^ |
| COL4A3 | 0.084 ^NS^ | 0.015^*^ | 0.148 ^NS^ | 0.002^**^ | 0.094 ^NS^ |
| COL5A1 | 0.002^**^ | 0.032^*^ | 0.008^**^ | 0.001^**^ | 0.012^*^ |
| COL5A2 | 0.209 ^NS^ | 0.282 ^NS^ | 0.241 ^NS^ | 0.031^*^ | 0.371 ^NS^ |
| COL5A3 | 0.305 ^NS^ | 0.010^*^ | 0.070 ^NS^ | 0.012^*^ | 0.809 ^NS^ |
| CSF2 | 0.052 ^NS^ | 0.009^**^ | 0.006^**^ | 0.001^**^ | 0.435 ^NS^ |
| CSF3 | 0.068 ^NS^ | 0.225 ^NS^ | 0.262 ^NS^ | 0.002^**^ | 0.183 ^NS^ |
| CTGF | 0.010^*^ | 0.009^**^ | 0.023^*^ | 0.008^**^ | 0.040^*^ |
| CTNNB1 | 0.027^*^ | 0.770 ^NS^ | 0.245 ^NS^ | 0.002^**^ | 0.093 ^NS^ |
| CTSG | 0.136 ^NS^ | 0.015^*^ | 0.212 ^NS^ | 0.001^**^ | 0.687 ^NS^ |
| CTSK | 0.136 ^NS^ | 0.410 ^NS^ | 0.176 ^NS^ | 0.021^*^ | 0.687 ^NS^ |
| CXCL11 | 0.018^*^ | 0.015^*^ | 0.012^*^ | 0.764 ^NS^ | 0.022^*^ |
| CXCL12 | 0.028^*^ | 0.020^*^ | 0.018^*^ | 0.001^**^ | 0.015^*^ |
| CXCL5 | 0.384 ^NS^ | 0.361 ^NS^ | 0.323 ^NS^ | 0.010^*^ | 0.074 ^NS^ |
| DCN | 0.043^*^ | 0.583 ^NS^ | 0.017^*^ | 0.007^**^ | 0.015^*^ |
| EGF | 0.028^*^ | 0.016^*^ | 0.015^*^ | 0.010^*^ | 0.015^*^ |
| EGFR | 0.503 ^NS^ | 0.076 ^NS^ | 0.212 ^NS^ | 0.001^**^ | 0.757 ^NS^ |
| F13A1 | 0.008^**^ | 0.041^*^ | 0.212 ^NS^ | 0.002^**^ | 0.022^*^ |
| F3 | 0.145 ^NS^ | 0.057 ^NS^ | 0.275 ^NS^ | 0.030^*^ | 0.659 ^NS^ |
| FGA | 0.186 ^NS^ | 0.009^**^ | 0.039^*^ | 0.003^**^ | 0.072 ^NS^ |
| FGF10 | 0.023^*^ | 0.010^*^ | 0.018^*^ | 0.865 ^NS^ | 0.012^*^ |
| FGF2 | 0.064 ^NS^ | 0.024^*^ | 0.003^**^ | 0.003^**^ | 0.026^*^ |
| FGF7 | 0.031^*^ | 0.024^*^ | 0.009^**^ | 0.076 ^NS^ | 0.064 ^NS^ |
| HBEGF | 0.251 ^NS^ | 0.587 ^NS^ | 0.059 ^NS^ | 0.012^*^ | 0.180 ^NS^ |
| HGF | 0.027^*^ | 0.003^**^ | 0.021^*^ | 0.002^**^ | 0.008^**^ |
| IFNG | 0.007^**^ | 0.009^**^ | 0.000^***^ | 0.006^**^ | 0.012^*^ |
| IGF1 | 0.006^**^ | 0.010^*^ | 0.006^**^ | 0.002^**^ | 0.012^*^ |
| IL10 | 0.006^**^ | 0.002^**^ | 0.002^**^ | 0.011^*^ | 0.064 ^NS^ |
| IL1B | 0.028^*^ | 0.198 ^NS^ | 0.118 ^NS^ | 0.871 ^NS^ | 0.116 ^NS^ |
| IL2 | 0.001^**^ | 0.002^**^ | 0.003^**^ | 0.012^*^ | 0.022* |
| IL4 | 0.007^**^ | 0.002^**^ | 0.002^**^ | 0.000^***^ | 0.022^*^ |
| IL6 | 0.028^*^ | 0.035^*^ | 0.042^*^ | 0.009^**^ | 0.012^*^ |
| IL6ST | 0.292 ^NS^ | 0.027^*^ | 0.044^*^ | 0.771 ^NS^ | 0.116 ^NS^ |
| ITGA1 | 0.008^**^ | 0.008^**^ | 0.004^**^ | 0.002^**^ | 0.053 ^NS^ |
| ITGA2 | 0.186 ^NS^ | 0.016^*^ | 0.318 ^NS^ | 0.008^**^ | 0.687 ^NS^ |
| ITGA3 | 0.029^*^ | 0.002^**^ | 0.001^**^ | 0.001^**^ | 0.022^*^ |
| ITGA4 | 0.030^*^ | 0.015^*^ | 0.006^**^ | 0.001^**^ | 0.033^*^ |
| ITGA5 | 0.068 ^NS^ | 0.066 ^NS^ | 0.035^*^ | 0.014^*^ | 0.862 ^NS^ |
| ITGA6 | 0.292 ^NS^ | 0.091 ^NS^ | 0.377 ^NS^ | 0.854 ^NS^ | 0.208 ^NS^ |
| ITGAV | 0.229 ^NS^ | 0.587 ^NS^ | 0.377 ^NS^ | 0.010^*^ | 0.918 ^NS^ |
| ITGB1 | 0.305 ^NS^ | 0.516 ^NS^ | 0.261 ^NS^ | 0.030^*^ | 0.912 ^NS^ |
| ITGB3 | 0.012^*^ | 0.002^**^ | 0.003^**^ | 0.010^*^ | 0.028^*^ |
| ITGB5 | 0.226 ^NS^ | 0.015^*^ | 0.106 ^NS^ | 0.010^*^ | 0.703 ^NS^ |
| ITGB6 | 0.028^*^ | 0.002^**^ | 0.005^**^ | 0.001^**^ | 0.015^*^ |
| LIF | 0.006^**^ | 0.014^*^ | 0.003^**^ | 0.002^**^ | 0.012^*^ |
| MAPK1 | 0.174 ^NS^ | 0.361 ^NS^ | 0.176 ^NS^ | 0.018^*^ | 0.659 ^NS^ |
| MAPK3 | 0.030^*^ | 0.002^**^ | 0.042^*^ | 0.011^*^ | 0.008^**^ |
| MMP1 | 0.142 ^NS^ | 0.005^**^ | 0.062 ^NS^ | 0.005^**^ | 0.801 ^NS^ |
| MMP2 | 0.045^*^ | 0.038^*^ | 0.066 ^NS^ | 0.002^**^ | 0.040^*^ |
| MMP7 | 0.018^*^ | 0.012^*^ | 0.005^**^ | 0.003^**^ | 0.034^*^ |
| MMP9 | 0.028^*^ | 0.015^*^ | 0.001^**^ | 0.002^**^ | 0.015^*^ |
| PDGFA | 0.176 ^NS^ | 0.029^*^ | 0.044^*^ | 0.970 ^NS^ | 0.801 ^NS^ |
| PLAT | 0.023^*^ | 0.003^**^ | 0.002^**^ | 0.014^*^ | 0.008^**^ |
| PLAU | 0.030^*^ | 0.009^**^ | 0.004^**^ | 0.002^**^ | 0.371 ^NS^ |
| PLAUR | 0.016^*^ | 0.002^**^ | 0.005^**^ | 0.003^**^ | 0.001^**^ |
| PLG | 0.006^**^ | 0.002^**^ | 0.000^***^ | 0.006^**^ | 0.012^*^ |
| PTEN | 0.219 ^NS^ | 0.587 ^NS^ | 0.235 ^NS^ | 0.970 ^NS^ | 0.766 ^NS^ |
| PTGS2 | 0.098 ^NS^ | 0.119 ^NS^ | 0.290 ^NS^ | 0.119 ^NS^ | 0.024^*^ |
| RAC1 | 0.198 ^NS^ | 0.298 ^NS^ | 0.380 ^NS^ | 0.046^*^ | 0.736 ^NS^ |
| RHOA | 0.251 ^NS^ | 0.015^*^ | 0.212 ^NS^ | 0.058 ^NS^ | 0.090 ^NS^ |
| SERPINE1 | 0.028^*^ | 0.008^**^ | 0.002^**^ | 0.001^**^ | 0.012^*^ |
| STAT3 | 0.229 ^NS^ | 0.173 ^NS^ | 0.113 ^NS^ | 0.030^*^ | 0.809 ^NS^ |
| TAGLN | 0.312 ^NS^ | 0.038^*^ | 0.030^*^ | 0.945 ^NS^ | 0.183 ^NS^ |
| TGFA | 0.318 ^NS^ | 0.571 ^NS^ | 0.051 ^NS^ | 0.021^*^ | 0.634 ^NS^ |
| TGFB1 | 0.018^*^ | 0.002^**^ | 0.004^**^ | 0.001^**^ | 0.012^*^ |
| TGFBR3 | 0.047 ^NS^ | 0.009^**^ | 0.108 ^NS^ | 0.001^**^ | 0.801 ^NS^ |
| TIAM1 | 0.322 ^NS^ | 0.075 ^NS^ | 0.149 ^NS^ | 0.691 ^NS^ | 0.801 ^NS^ |
| TIMP1 | 0.065 ^NS^ | 0.024^*^ | 0.121 ^NS^ | 0.012^*^ | 0.809 ^NS^ |
| TNF | 0.016^*^ | 0.012^*^ | 0.000^***^ | 0.001^**^ | 0.008^**^ |
| VEGFA | 0.179 ^NS^ | 0.029^*^ | 0.052 ^NS^ | 0.025^*^ | 0.279 ^NS^ |
| VTN | 0.011^*^ | 0.008^**^ | 0.012^*^ | 0.003^**^ | 0.001^**^ |
| WISP1 | 0.003^**^ | 0.015^*^ | 0.004^**^ | 0.012^*^ | 0.008^**^ |
| WNT5A | 0.168 ^NS^ | 0.845 ^NS^ | 0.209 ^NS^ | 0.030^*^ | 0.027^*^ |

BH-FDR: Benjamini-Hochberg-False Discovery rate . *** p < 0.001; ** p < 0.01; * p < 0.05 and NS: Non-Sig
